# Psychometric properties of principals’ attitudes toward inclusive education (PATIE) scale: Arabic version

**DOI:** 10.1186/s40359-024-01524-z

**Published:** 2024-01-12

**Authors:** Mubarak S. Aldosari

**Affiliations:** Special Education Department, Prince Sattam Bin Abdulaziz University, Alkharj, Saudi Arabia

**Keywords:** Confirmatory factor analysis, Principals, Attitudes, Inclusion, Students with disabilities, PATIE scale

## Abstract

**Background:**

Inclusive education is critical for the successful integration of students with disabilities into general education schools, and principals’ attitudes play a crucial role in this process. Despite the recognized significance of attitudes, there remains a gap in understanding these attitudes among principals in Arabic-speaking regions concerning inclusive education practices. This study aims to bridge this gap by validating and assessing the reliability of the Arabic version of the Principals’ Attitudes Toward Inclusive Education (PATIE) scale.

**Methods:**

To measure these attitudes in the Arab region, the current study validated and assessed the reliability of the Arabic version of the Principals’ Attitudes Toward Inclusive Education (PATIE) scale using a sample of 391 principals from schools that have in place inclusion programs for students with disabilities. Confirmatory factor analysis (CFA) was employed to validate the scale’s structural, discriminant, and convergent validity, while Cronbach’s alpha and composite reliability (CR) were utilized to evaluate the scale’s reliability.

**Results:**

The results demonstrated the strong validity and reliability of the Arabic version of the PATIE, with all five factors displaying good reliability.

**Conclusions:**

These findings suggest that the scale can effectively measure attitudes toward inclusive education in Arabic-speaking countries. This study’s implications for research and practice are significant, as they underscore the importance of positive attitudes among principals in promoting inclusive education and provide a validated tool for measuring these attitudes.

## Introduction

Special education has undergone significant changes over the past 40 years, as indicated by an increase in the inclusion of students with disabilities in general education schools, across several countries [1]. Inclusive education refers to the practice of educating students with disabilities in general education classrooms or schools, providing them with equal opportunities and the same access to educational resources and services as that experienced by their peers without disabilities.

The significance of inclusive education cannot be overstated, as they provide a wide range of benefits to leaners, teachers, and society as a whole. Inclusive approach of education facilitates equity and celebrate diversity by bringing together students from a variety of backgrounds and with different abilities and perspectives. Inclusive education is also helpful for improvements in the academic, functional, and social communication skills of students with disabilities, as well as the acquisition of positive behaviors [2–5]. Additionally, the inclusion of students with disabilities has helped improve the negative stereotyped perceptions harbored by students without disabilities toward their peers with disabilities [6].

The success of inclusion programs for students with disabilities depends on various factors, including the qualifications and abilities of the staff working in these programs [7, 8], as well as the attitudes of the staff toward inclusion [9, 10]. School principals’ attitudes are particularly crucial to the success of inclusion programs for students with disabilities, as they play a vital role in developing and implementing inclusive policies and practices in schools [11].

School principals themselves are also essential to the success of inclusion programs for students with disabilities in general schools, especially when it comes to developing and achieving the school’s vision and goals, creating a collaborative and welcoming environment for school staff, promoting positive attitudes toward the inclusion of students with disabilities, and supporting the education of all students in the school. Furthermore, they are key personnel to establish a supportive and inclusive school culture, offering leadership, and ensuring that policies and practices are consistent with the principles of inclusion [12, 13].

Therefore, examining school principals’ attitudes toward inclusion is critical when it comes to the success of inclusion programs for students with disabilities. Principals serve as the leaders who set the tone for the school’s culture and policies, and their attitudes can either facilitate or hinder the implementation of inclusive education. As alluded to above, school principals’ attitudes have a significant impact on the success of inclusion programs for students with disabilities; this is due to the fact that attitudes are crucial to behavioral intentions [10]. Principals with positive attitudes toward inclusion are more likely to implement inclusive policies and practices in schools, promote a welcoming and inclusive environment for all students, and support staff in developing their abilities and skills to work with students with disabilities. Understanding school principals’ attitudes provides valuable insights into their commitment to creating an inclusive learning environment where all students, regardless of their abilities or backgrounds, have equal access to quality education. By assessing principals’ attitudes, educational stakeholders can identify areas where professional development and support may be needed, ensure alignment with inclusive education policies, and ultimately foster an environment that prioritizes the inclusion, diversity, and equitable opportunities necessary for the academic and social growth of all students.

One helpful instrument for assessing principals’ attitudes toward inclusion is the Principals’ Attitudes Toward Inclusive Education (PATIE) scale developed by Bailey [14]. The PATIE scale stands as a tool aligned with overarching theoretical viewpoints on educational leadership. Extensive research underscores the pivotal role of inclusive leadership in establishing an environment that embraces and supports all students [15]. This scale serves as a valuable instrument in gauging the attitudes of school principals—a critical element in comprehending the leadership’s role in nurturing inclusive education [15]. Furthermore, inclusive leadership emerges as a mediating force, influencing the correlation between educators’ work engagement and their innovative behaviors [16]. This underscores the profound significance of leadership in championing inclusive practices within educational settings.

Moreover, the scale resonates with broader theoretical frameworks on inclusion. Studies substantiate that principals’ attitudes wield substantial influence on the success of inclusive education, particularly for students with disabilities. Positive attitudes among school leaders correlate with enhanced academic performance, emphasizing the critical role attitudes play in fostering inclusion [16]. By offering a means to measure these attitudes, the PATIE scale contributes significantly to unraveling the intricacies of factors impacting the effective implementation of inclusive education. It serves as a beacon, illuminating the pathways toward creating inclusive educational environments that cater to the diverse needs of all learners.

While instruments tailored explicitly to principals and their roles as instructional and special education leaders are lacking, the PATIE scale has been used in various countries, and various investigations, to assess principals’ attitudes toward inclusion [17]; this encompasses both US-based research [18–22] and studies conducted in other countries, such as the Philippines [21], Turkey [17, 23], South Korea [24], and Hong Kong [25]. The structure of the PATIE scale shows a high level consistency across different cultures. Previous researches have demonstrated the robustness of the scale’s overall structure, indicating that the underlying dimensions remain conceptually stable across different settings [17, 18, 23, 26]. The factorial structure of the PATIE scale, as evidenced in previous studies, typically reveals a multidimensional construct representing principals’ attitudes toward inclusive education. Across various cultural contexts, the scale commonly exhibits a factor-based structure encompassing multiple dimensions that capture distinct aspects of attitudes pertinent to inclusive educational practices [26].

A considerable number of studies have chosen not to examine the reliability and validity of the PATIE scale, instead relying on the psychometric properties established by Baily’s initial study sample (e.g., [19, 24, 25]). However, some researchers have evaluated the reliability and validity of the PATIE scale within specific participant groups and countries. Nguluma [17] investigated the psychometric properties of the PATIE scale in Turkey’s Sakarya district and found that Cronbach’s alpha coefficient was 0.78. Similarly, Boston [18] demonstrated the validity and reliability of the PATIE scale in New Hampshire, USA. These high levels of internal consistency suggest that the PATIE scale is a reliable instrument for measuring principals’ attitudes toward inclusion.

It is also important to consider the limitations of the scale’s generalizability. The samples used in these studies were specific to certain regions and populations, and the psychometric properties of the scale in Arabic have not been examined. Given the relative novelty of inclusive education in the Arab region, developing a validated Arabic version of the PATIE scale would be a valuable step for researchers and practitioners seeking to promote inclusive education for students with disabilities. A psychometrically-sound Arabic PATIE scale would benefit both inclusive education studies and the field, allowing international data comparisons and ensuring cross-cultural validity. Our study aims to evaluate the validity and reliability of the Arabic version of the PATIE scale, specifically investigating principals’ attitudes toward the inclusion of students with disabilities in the Arab region.

## Methods

### Sample characteristics

This study focuses on the principals of government schools offering special education programs during the 2022–2023 academic year in Riyadh, Saudi Arabia. Riyadh was selected because of its high number of schools with special education programs, specifically 838 education programs for students with disabilities attached to 600 schools [27]. Riyadh is the capital and one of the largest cities in Saudi Arabia. Research conducted in Riyadh can offer insights that may be applicable to a broader context within the country due to its diverse population and representation of various socioeconomic backgrounds. Additionally, Riyadh often serves as a focal point for the implementation of educational policies and initiatives in Saudi Arabia. By focusing on government schools in Riyadh, where special education programs are being implemented, the study can provide valuable data that could influence future policies regarding inclusive education across the country. It is important to note that the school system in Saudi Arabia is divided by gender, with female and male principals in female and male schools, respectively.

All principals of schools offering programs for students with disabilities were included in the study, resulting in a total participant pool of 600 principals who completed the survey. Of the total respondents, 391 principals provided usable data for statistical analysis, yielding a response rate of 65.16%. This sample size, exceeding the usual minimum of 200 respondents recommended for factor analysis [28], was considered adequate for CFA, as there were more than ten participants [29]. However, it should be noted that there is ongoing debate regarding the appropriate sample size for factor analysis.

Table 1 presents the demographic data of the 391 respondents. The survey was conducted with school principals of all genders, and the majority—albeit a slim one—of the respondents identified as male (54%). Respondents were principals from elementary (50.6%), middle (33.3%), and high schools (16.1%). Among the schools represented in this study, 23.5% provided special education programs for students with intellectual disabilities. In comparison, 19.18, 16.62, 15.1, 7.40, and 10% of these schools offered programs for students with autism, learning disabilities, deafness or difficulty hearing, visual impairment, and multiple disabilities, respectively.

**Table 1:** Demographic characteristics of school principals

Variables		Frequency	Percentage
Gender	Male	211	54
	Female	180	46
Level of school	Elementary	198	50.6
	Middle	130	33.3
	High school	63	16.1
Type of special education program	Intellectual Disability	104	23. 5
	Autism	80	19.18
	Learning Disability	69	16.62
	Deaf/Hard of Hearing	54	15.1
	Visual impairment	31	7.4
	Multiple disabilities	43	10

The age range of the participants was 30–60 years, and their educational attainment varied. Among them, 96 (24.5%) were between 30 and 40 years old, 169 (43.3%) were between 40 and 50 years old, and 126 (32.3%) were between 50 and 60 years old. In terms of educational attainment, 322 respondents (82.4%) held a bachelor’s degree, 61 (15.6%) held a master’s degree, and eight (2%) held a doctoral degree.

Overall, the sample of 391 principals from Riyadh offers a comprehensive representation of the broader principal population in the region, ensuring that the statistical analysis is valid and reliable.

### Instrument

The PATIE scale was developed to evaluate principals’ attitudes toward the inclusion of students with disabilities in general schools. The original survey contained 30 items. However, Bailey (2004) modified the scale in 2004, with the new version containing 24 items divided into five factors:

- Factor 1: Teacher Workload and Management (TWM) (five items, e.g., “Including students with special needs creates few additional problems for teachers’ class management”)
- Factor 2: Inclusion Benefits and Levels of Disability (IBLD) (six items, e.g., “Students with mild disabilities should be included in regular classrooms”)
- Factor 3: Learning Challenges in Inclusive Settings (LCIS) (seven items, e.g., “Students who cannot read normal print size should not be included in regular classrooms”)
- Factor 4: Excluded Students (ES) (three items, e.g., “Students with severe disabilities should be included in regular classrooms”)
- Factor 5: Professional Training (PT) (three items, e.g., “Teacher aides are trained adequately to cope with students with special needs”)

The PATIE scale, consisting of 24 items, with 14 negatively- and 10 positively-phrased statements, utilizes a five-point Likert format that spans from 1 (strongly disagree) to 5 (strongly agree). Higher scores signify more positive attitudes toward the inclusion of students with disabilities in mainstream educational environments [14]. The internal consistency of the scale, as measured using Cronbach’s alpha, was 0.92.

Overall, the PATIE scale has proven to be a reliable and widely employed tool for assessing principals’ attitudes towards the inclusion of students with disabilities in general education settings. Its five-factor structure enables more detailed insights into principals’ attitudes, while the use of both positively- and negatively-worded items aids in mitigating response bias.

### Translation of the PATIE scale

Translation of the scale from English to Arabic was the first step in testing its validity and reliability in an Arab context. We initially obtained permission from Jeff Bailey to use the PATIE scale. The translation process involved several stages and adopted a committee approach [30]. Bilingual experts who are associate professors in the English Language department of [anonymized for review] University translated the English version of the instrument into Arabic. The professors hold Ph.D. in English linguistic and were native Arabic speakers. Subsequently, a different bilingual individual who is an associate professor at the University translated the Arabic version into English without consulting the original text. The back-translated version was compared to the original English version to ensure the accuracy of the Arabic translation. This step added an extra layer of validation to the translation process. The translation team discussed and resolved any issues to confirm that the translated version accurately represented the original PATIE. This thorough translation and back-translation process aimed to strengthen the reliability and validity of the Arabic version of the PATIE scale, ensuring that it effectively measured principals’ attitudes toward the inclusion of students with disabilities in general education schools in the Arab world. As an additional layer of quality translation, the translated scale was reviewed by experts in the field to ensure linguistic accuracy and cultural relevance.

### Procedures

To maintain ethical and regulatory adherence, the researcher sought prior approval from an Institutional Review Board and the Ministry of Education. Once approval had been granted, the researcher acquired the contact information of the principals of the 600 schools from the Ministry of Education, and initiated the survey.

The participants were contacted via email and text message and provided with detailed information about the purpose of the study and procedures for completing the survey. Prior to the survey, all respondents consented to participate in the study. The survey was completed in 2 weeks and eight surveys (2% of the sample) were excluded from the analysis because of factors such as duplicate submissions, participants not meeting the eligibility criteria, or responses that fell outside the predetermined timeframe. As an additional measure to ensure the content validity of the Arabic version, the scale went through a thorough review by a panel of experts in the field of inclusive education, educational psychology, and scale development to validate the relevance and representativeness of the items in the constructs.

### Data management and analysis

To assess the reliability and validity of the Arabic version of the PATIE scale, the researcher used the Statistical Package for Social Sciences (SPSS) version 25 and IBM SPSS Amos 27 to perform CFA and calculate Cronbach’s alpha coefficient for internal consistency. A sample of 391 participants was deemed sufficient for the CFA [31]. The researchers also tested for random distribution of missing data (*p* > 0.05) using Little’s test [32], and missing data were accounted for using AMOS when carrying out the analysis. CFA was conducted with the incorporation of missing data, given that the data were missing completely at random (MCAR) [33].

The descriptive statistics for the PATIE scale items reveal a range of patterns in the responses (see Table 2). The means of the items vary from 2.62 (P21) to 4.01 (P24), indicating a spread in the central tendency across different items. The standard deviations, ranging from 0.813 (P13) to 1.394 (P18), suggest varying degrees of variability in responses, with some items showing more consensus among respondents than others. Notably, the skewness values, which range from − 1.831 (P13) to 0.243 (P21), highlight that most items are negatively skewed, indicating a tendency for respondents to score towards the higher end of the scale. However, a few items like P18 and P21 show a near-zero or slightly positive skewness, suggesting a more symmetric distribution or a slight tendency towards lower scores. The kurtosis values, ranging from − 1.202 (P18) to 2.652 (P13), vary significantly, with some items showing a platykurtic distribution (flatter than a normal distribution) and others a leptokurtic one (more peaked). This diversity in skewness and kurtosis across items suggests differing levels of extremity in responses, with some items eliciting more moderate responses and others more extreme ones. It is important to mention that skewness and kurtosis values for all 24 items were under acceptable range, suggesting data normality (see Table 2 for details).

**Table 2:** Descriptive statistics of scale items

Item	Mean	Std. Error	Std. Deviation	Skewness	Kurtosis
P3	3.53	0.059	1.160	−0.686	− 0.134
P10	3.08	0.063	1.240	−0.397	− 0.702
P16	3.85	0.060	1.193	−0.944	0.088
P21	2.62	0.064	1.262	0.243	−1.025
P28	2.92	0.065	1.286	−0.147	−1.069
P9	3.22	0.069	1.361	−0.448	−0.933
P11	3.23	0.060	1.184	−0.414	−0.541
P15	3.50	0.065	1.282	−0.763	−0.306
P25	3.24	0.061	1.198	−0.535	−0.488
P26	3.40	0.061	1.208	−0.622	− 0.348
P27	3.59	0.056	1.104	−0.719	− 0.015
P2	3.09	0.067	1.315	−0.250	− 1.064
P4	3.92	0.060	1.176	−1.052	0.395
P5	3.01	0.058	1.148	−0.384	−0.766
P12	3.62	0.058	1.152	−0.777	0.018
P18	2.84	0.071	1.394	0.087	−1.202
P19	3.56	0.064	1.257	−0.734	−0.390
P29	3.23	0.065	1.279	−0.367	−0.805
P6	3.85	0.057	1.125	−1.072	0.586
P17	3.75	0.058	1.153	− 1.034	0.471
P24	4.01	0.057	1.120	−1.147	0.734
P1	3.89	0.059	1.161	−1.031	0.357
P13	3.53	0.041	0.813	−1.831	2.652
P20	3.25	0.068	1.347	−0.477	−0.938

Overall, these statistics indicate a complex pattern of responses across the PATIE scale. Furthermore, prior to data analysis, data normality was assessed. A multivariate Normality test was conducted in the AMOS. The results indicated that the critical ratio (c.r.) of multivariate normality for most of the items is below the threshold of ±6 as recommended by Yuan, Bentler and Zhang (2005). All the skewness values for 24 items were ranging from − 1.82 to 0.24 with the average critical ration (CR) value of 5.12. Whereas, all Kurtosis values were ranging from − 1.20 to 2.60 with the average CR value of 0.96. Moreover, when the sample size is high then data is close to normality distribution regardless of the distribution of population. According to the Central Limit Theorem (CLT) the distribution of sample means approximates a normal distribution regardless of the population’s distribution as the sample size gets larger (Stark, 2017).

To test the scale data’s conformity to the CFA model, the researcher employed multiple goodness-of-fit indices, including the Comparative Fit Index (CFI), the Tucker-Lewis Index (TLI), Root Mean Square Error of Approximation (RMSEA), the Standardized Root Mean Square Residual (SRMR) Index, and the ratio of the minimum variance function to degrees of freedom (CMIN/DF < 3.0). Acceptable CFI values are greater than or equal to 0.90, whereas values exceeding 0.95 signify a good model fit [34]. For RMSEA, values below 0.08 denote acceptable fit, while those below 0.05 indicate good model fit [28, 35]. SRMR values under 0.08 are considered acceptable, while those under 0.05 represent a good model fit [34, 36].

The researcher used a first-order CFA model to verify the correlation between the latent variables represented in the five factors of the Arabic version of the PATIE scale (TWM, IBLD, LCIS, ES, and PT), while a second-order CFA model was used to test the conformity of the factors of the scale (TWM, IBLD, LCIS, ES, and PT) to a common higher-order factor. After evaluating the model fit, composite reliability (CR) and average variance extracted (AVE) were calculated to verify the convergent and discriminant validity of the Arabic version of the PATIE scale.

Convergent validity (CV ≥ 0.5) was established by verifying the factor loadings of the indicators, CR (≥ 0.7), and AVE (≥ 0.5) [37]. Discriminant validity (DV) was confirmed using two widely-accepted methods. The first method involved ensuring that the correlation coefficient between two potential variables did not exceed 0.9, as a value greater than 0.9 indicates substantial overlap between the variables [31]. The second method involved comparing the AVE with the squared value of the correlation coefficient between two latent variables, such that the square root of the AVE is greater than the correlation coefficient between the latent variables [38, 39].

Finally, the reliability of the Arabic version of the PATIE scale was assessed by computing Cronbach’s alpha coefficient for each factor individually. An alpha value of 0.70 or higher was established as the threshold for acceptable internal consistency of the scale [40, 41].

## Results

### Confirmatory factor analysis (CFA)

CFA was utilized to evaluate the factorial validity of the Arabic version of the PATIE scale. Table 3 displays the conformity indicators for the single-factor and five-factor models with first and second order. The findings indicate that the single-factor model did not exhibit a satisfactory fit compared with the first- and second-order five-factor models. The indicators of goodness of fit for the single-factor model were as follows: χ2 = 961.729, *df* = 252; χ2*/df* = 3.82; CFI = 0.784; GFI = 0.817; RMSEA = 0.098 (CI_90%_ = 0.093–0.104); SRMR = 0.086. These values suggest a poor model fit. Specifically, the goodness-of-fit index (CFI) and GFI values were lower than the recommended cutoff score of 0.90 [34], indicating that the model did not fit well. The RMSEA value exceeded the suggested cutoff of 0.08 [35], again implying that the model did not fit the data well. Additionally, the SRMR values were higher than the recommended cutoff score of 0.08 [34], signaling that the model might not represent the data well. The findings suggest that the single-factor model was not a good fit for the data, whereas the five-factor model with first and second order had a better fit.

**Table 3:** Indicators of the goodness of fit of CFA models

Model	χ^2^	*df*	*χ*^*2*^*/df*	*CFI*	*GFI*	*RMSEA*	*SRMR*
						*CI 90%*	
One factor	961.73	252	3.82	0.78	0.82	0.098	0.086
						[.093–.104]	
Correlated five factors	526.11	242	2.17	0.96	0.94	0.065	0.057
						[.060–.071]	
Second-order factor	546.62	247	2.21	0.95	0.93	0.066	0.052
						[.059–.070]	

*df*, degrees of freedom, *RMSEA* Root mean square error of approximation, *GFI* Goodness of fit index, *CFI* Comparative fit Index

**Fig. 1:**
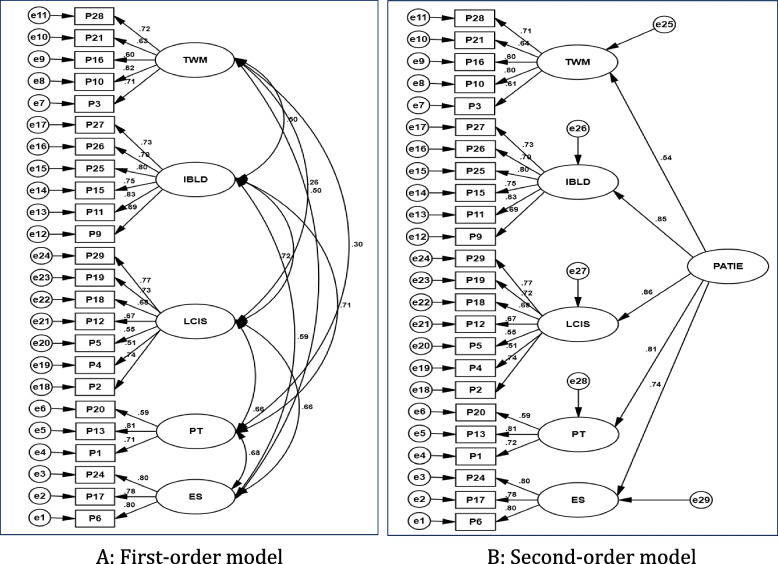
First and second-order CFA models. Note: TWM = Teacher Workload and Management; IBLD = Inclusion Benefits and Levels of Disability; LCIS = Learning Challenges in Inclusive Settings; ES = Excluded Students PT = Professional Training

Subsequently, the correlated five-factor model was examined. The results indicated that the correlated five-factor model provided a better fit than did the one-factor model. This improvement was supported by a significant decrease in the chi-squared value and an increase in goodness-of-fit indices such as CFI, GFI, RMSEA, and SRMR. All indicators of goodness of fit were considered acceptable, with χ^2^(242) = 526.1 and χ2*/df* = 2.17. The CFI and GFI values were 0.961 and 0.938, respectively, whereas the RMSEA and SRMR values were 0.065 (CI_90%_ = 0.060–0.071) and 0.057, respectively. The Akaike Information Criterion (AIC) was used to confirm the goodness-of-fit of the correlated five-factor model. The AIC value of the first-order five-factor model was 341.362, which was lower than the AIC value of the one-factor model (AIC = 528.448). This suggests that the correlated five-factor model provided a better fit to the data. The chi-squared difference test was used to compare the fit of the correlated five- and one-factor models. The results indicate that the correlated five-factor model provided a better fit than did the one-factor model (Δχ^2^_(10)_ = 435.619, *p* < 0.001). The second-order five-factor model also exhibited better performance than did the one-factor model (Δχ^2^_(5)_ = 415.109, *p* < 0.001). Figure 1 shows the first- and second-order CFA of the PATIE scale with standardized factor loadings. The factor loadings for all items were greater than 0.50 and were significant at *p* < 0.01. This suggests that the items are reliable measures of the underlying constructs.

The reliability of the Arabic version of the PATIE scale was assessed using Cronbach’s alpha measure and as well with McDonald’s omega, with the results demonstrating an acceptable degree of internal consistency for all factors. Specifically, the Cronbach’s alpha reliability coefficients for the factors of the Arabic version of the PATIE scale were as follows: a) TWM; α = 0.823; b) IBLD; α = 0.880); c) LCIS; α = 0.849, d) ES; α = 0.836; and e) PT; α = 0.702). All Cronbach’s alpha values were greater than 0.7, which is considered acceptable and indicates that the Arabic version of the PATIE has good internal consistency.

Additionally, McDonald’s omega for estimating reliability was also used in the presence of non-uniform factor loadings. The McDonald’s omega coefficients for each subscale were as follows: a) TWM; ω = 0.77; b) IBLD; ω = 0.88); c) LCIS; ω = 0.83, d) ES; ω = 0.83; and e) PT; α = 0.65). Overall scale’s McDonald Omega was 0.91. These omega coefficients, reflecting a range from acceptable to excellent reliability, address the concern raised regarding the appropriateness of the reliability measure given the variability in item-factor loadings. The use of McDonald’s omega provides a more nuanced and accurate estimate of the internal consistency of the scale, taking into consideration the heterogeneity in factor loadings. This methodological adjustment strengthens the reliability analysis of the scale and enhances the robustness of the study’s findings.

Table 4 displays the standardized factor loadings for the items of the Arabic version of the PATIE scale and Cronbach’s alpha values. The loading values for the factors of the Arabic version of the PATIE scale on the second-order CFA model were found to be satisfactory, with a range of 0.51 for the LCIS factor to 0.83 for the IBLD factor.

**Table 4:** PATIE Arabic version’s loadings, reliability, and validity

Factor	Indicator		Estimate		Reliability		*AVE*
			*l*	*SE*	*a*	*CR*	
Teacher Workload and Management (TWM)	P3	Including students with special needs creates few additional problems for teachers’ class management	.712	.055	.823	.827	.500
	P10	Students with special needs will take up too much of the teacher aides’ time	.816	.058			
	P16	Regular students will be disadvantaged by having special needs children in their classrooms	.604	.060			
	P21	Including students with special needs is unfair to regular teachers who already have a heavy workload	.631	.062			
	P28	Students with special needs will take up too much of the teachers’ time	.721	.061			
Inclusion Benefits and Level of Disability (IBLD)	P9	Students with mild disabilities should be included in regular classrooms	.687	.063	.880	.884	.561
	P11	Regardless of whether the parents of regular students object to inclusion, the practice should be supported	.831	.050			
	P15	Students with disabilities benefit academically from inclusion	.749	.057			
	P25	Students with moderate disabilities should be included in regular classrooms	.796	.052			
	P26	Students with disabilities benefit socially from inclusion	.696	.055			
	P27	Regular students benefit socially from inclusion	.726	.050			
Learning Challenges in Inclusive Settings (LCIS)	P2	Students with physical disabilities (wrist crutches, wheelchairs) create too many movement problems to permit inclusion	.737	.060	.849	.848	.628
	P4	Students who cannot read normal print size should not be included in regular classrooms	.510	.059			
	P5	Because special schools are better resourced to cater to special needs students, these students should stay in special schools	.548	.057			
	P12	Special needs students belong in special schools where all their needs can be met	.675	.054			
	P18	Special needs students whose achievement levels in basic skills are significantly lower than their classmates (of the same age) should not be included in regular classrooms	.684	.065			
	P19	Students who have to communicate in a special way (e.g., communication boards/signing) should not be included in regular classrooms	.725	.057			
	P29	Students with severe speech difficulties should not be included in regular classrooms	.766	.057			
Excluded Students (ES)	P6	Students who are continually aggressive toward their fellow students should not be included in regular classrooms	.802	.051	.836	.837	.631
	P17	Students who are continually aggressive toward school staff should not be included in regular classrooms	.779	.052			
	P24	Students with severe disabilities should not be included in regular classrooms	.801	.050			
Professional Training (PT)	P1	Regular teachers are not trained adequately to cope with students with disabilities	.711	.056	.702	.752	.507
	P13	Teacher aides are trained adequately to cope with students with special needs	.814	.038			
	P20	Regular school principals are trained adequately to cope with students with disabilities	.593	.068			

*l* Standardized loadings, *AVE* Average Variance Extracted. a: Cronbach’s alpha. Composite Reliability (CR)

### Discriminant validity (DV)

Discriminant Validity is a vital aspect of validity in research, as it evaluates the degree to which measures of different constructs are distinct from each other and do not overlap. In other words, it refers to the extent to which a measure does not correlate strongly with measures of different constructs or concepts that are theoretically distinct [42, 43]. To rigorously assess the discriminant validity (DV) of the PATIE scale, several measures were employed, including the calculation of Composite Reliability (CR) and Average Variance Extracted (AVE), along with correlation analysis between subscales of the PATIE and their respective AVE. The CR provides a measure of the internal consistency of a scale by estimating the proportion of true variance to the total variance in the indicators. AVE measures the amount of variance captured by a construct relative to the measurement error [38, 44]. Table 5 presents the results of the CR and AVE analyses of the scale indicators. The AVE values range from 0.500 to 0.631, signaling that the indicators are consistent with the respective factors. In addition, the CR values range from 0.752 to 0.884, suggesting a high level of internal consistency within the PATIE scale. These results demonstrate that the PATIE factors measure different and distinct constructs, thereby supporting the DV of the scale.

**Table 5:** Correlation matrix with AVE and squared correlation values

	TWM	IBLD	LCIS	ES	PT
TWM	***0.5***	*0.249*	*0.249*	*0.068*	*0.089*
IBLD	**0.499**	***0.561***	*0.523*	*0.346*	*0.503*
LCIS	**0.501**	**0.723**	***0.628***	*0.436*	*0.436*
ES	**0.261**	**0.588**	**0.66**	***0.631***	*0.466*
PT	**0.299**	**0.709**	**0.66**	**0.683**	***0.507***

The average variance extracted (AVE) is denoted by bold and italicized values. Correlation is represented by italicized values above the diagonal of the matrix, while squared correlation is represented by bold values below the diagonal

Table 5 presents the correlation coefficients between the scale indicators. The values range from 0.26 to 0.72, all of which are below the threshold of 0.85, indicating good DV. The findings suggest that the PATIE scale’s indicators measure distinct constructs and do not overlap with each other. These result also confirm that the participants clearly and explicitly understood the indicators’ meanings, further supporting the DV of the scale.

To explore discriminant validity more comprehensively, we conducted further analyses focusing on factor inter-correlations. This allowed us to assess the relationships between factors and their respective Average Variance Extracted (AVE). Our analysis uncovered correlations among the factors, yet these correlations were notably lower than the square roots of the AVE for each factor. This crucial finding signifies that the variance shared between factors is substantially less than the variance explained by each individual factor. Essentially, these results provide compelling evidence that the factors assessed by the PATIE scale encapsulate distinct and unique constructs. The lower correlations among factors compared to the variance explained by each factor itself strongly support the scale’s discriminant validity. This robust evidence reinforces the notion that each factor measured by the scale indeed represents a separate and identifiable construct, affirming the scale’s credibility in assessing diverse aspects within the context of inclusive leadership. Based on the results presented above, it is evident that the findings of the CFA analysis of the PATIE scale align with those of the English version of the scale, thus providing strong evidence that the PATIE scale comprises five distinct factors. Overall, researchers can confidently use the scale to measure the constructs of interest without the risk of conflating or overlapping them.

## Discussion

This study aimed to establish the validity of the Arabic version of the PATIE scale, which is essential for facilitating comparisons of data on principals’ attitudes toward the inclusion of students with disabilities. Such data are critical for policymakers, agency administrators, and community workgroups seeking to improve the quality of education for students with disabilities [45]. By examining such data, it is possible to better understand what factors influence principals’ attitudes and how different demographic characteristics correlate with more positive attitudes.

The current study found that the Arabic version of the PATIE scale has good construct validity. CFA revealed that the five-factor model of the scale had a better fit for the data than did the one-factor model. The strong validity and reliability evidenced by the PATIE scale in this study are consistent with prior research conducted in different cultural contexts [15, 16]. The aforementioned factors have significant implications for the policymakers and practitioners involved in promoting inclusive education. For example, knowledge of the factors that influence principals’ attitudes towards inclusive education can help develop policies and interventions that address the challenges faced in inclusive settings. The factors identified in this study, such as TWM, can be used to develop targeted interventions to alleviate workload and management issues in inclusive schools. Similarly, knowledge of the benefits of inclusion, as well as the challenges and training needs associated with it, can aid in the development of evidence-based strategies to support students with disabilities in inclusive settings.

The high level of internal consistency found in this study provides strong evidence for the reliability of the Arabic version of the PATIE scale. Our findings’ consistency with those of previous research conducted in other countries, such as Chandler [19] in the US and Nguluma [23] and Nguluma et al. [15] in Turkey, further supports the reliability of the scale. Indeed, the reliability of a scale is a crucial aspect of its psychometric properties, as it determines the extent to which a scale can produce consistent and accurate results. The high level of internal consistency found in our study, and in previous research, supports the use of the Arabic version of the PATIE scale as a reliable and valid measure in Arabic-speaking regions. However, the present study extends this understanding by highlighting the distinctive nature of these factors, reinforcing their ability to capture diverse attitudes rather than overlapping constructs. This aligns with recent researches [45, 46], emphasizing the necessity of assessing not only internal consistency but also discriminant validity to ensure a comprehensive evaluation of measurement tools.

By validating the PATIE scale in Arabic, this study expands existing knowledge based on the psychometric properties of the scale and strengthens its utility as a valuable tool for assessing attitudes toward inclusive education in Arabic-speaking countries. The findings can inform the development of evidence-based policies and interventions aimed at addressing the challenges encountered in inclusive settings.

## Implications for research and practice

The study has significant implications for future research and practices in inclusive education. Researchers can use the Arabic version of the PATIE scale as a valuable tool to with which measure school principals’ attitudes toward inclusion in Arabic-speaking countries. This tool can aid in collecting data for cross-cultural comparisons and in developing evidence-based interventions to address the challenges faced in inclusive settings. Policymakers can utilize this information to develop policies that support the implementation of inclusive education and to ensure that schools have the resources necessary to provide quality education to all students. Moreover, for practitioners, the five-factor model identified in this study can guide the development of targeted interventions to address the specific needs of inclusive schools.

Validation of the Arabic version of the PATIE scale represents a critical step toward promoting inclusive education for students with disabilities in Arabic-speaking regions. Future research should aim to validate the scale in other contexts and explore the attitudes of other stakeholders toward inclusion. In doing so, we can continue to promote inclusive education and ensure that all students receive the education they deserve.

Moreover, principals’ positive attitudes play an essential role in the inclusion of students with disabilities in general education schools. The Arabic version of the PATIE scale can be employed by researchers to provide an understanding of the potential variables that could assist in improving principals’ attitudes toward the inclusion of students with disabilities, and would also aid in providing data which officials can utilize to make changes to existing policies.

The Rasch analysis provides a unified approach for examining several measurement properties, allowing for a more comprehensive understanding of a scale’s construct validity [47]. Incorporating Rasch analysis into future studies, in addition to the classical CFA approach, could offer a more in-depth assessment of the psychometric properties of the PATIE scale, and could also inform further development and refinement of the scale.

## Limitations

This study has three notable limitations. First, the sample was drawn exclusively from Saudi Arabia, which may limit the generalizability of the results to other Arabic-speaking countries. Though by concentrating on Saudi Arabia, the present study can offer a comprehensive examination of how policies are translated into practices and attitudes within a specific national educational system, it calls for future research to replicate the study in other Arabic-speaking countries. This would allow for a more comprehensive understanding of how cultural and contextual differences may influence principals’ attitudes toward inclusive education.

Second, the current study only included principals from elementary, middle, and high school levels, with no focus on principals from kindergarten schools that enroll children with disabilities. Principals at the kindergarten level might have different attitudes toward the inclusion of students with disabilities – attitudes which may be influenced by factors such as the child’s age and developmental level. Therefore, future studies ought to include principals from kindergarten schools so as to provide a more comprehensive understanding of their attitudes toward the inclusion of students with disabilities at different developmental stages.

Finally, the current study only included schools whose students have various disabilities and excluded those with physical disabilities, as students with physical disabilities are often enrolled in special education programs rather than in general schools. However, it is essential to note that physical disabilities are also a significant aspect of disability inclusion, and principals’ attitudes toward including students with physical disabilities are equally important. Therefore, future studies ought to include the principals of schools that enroll students with physical disabilities, so that insights can be provided into how schools can better support such students and promote social inclusion in regular classrooms.

## Data Availability

The datasets used and/or analyzed during the current study are available from the corresponding author on reasonable request.

## Abbreviations

PATIE: Principals’ attitudes toward inclusive education
CFA: Confirmatory factor analysis
CR: Cronbach’s alpha and composite reliability
SPSS: Statistical package for social sciences
AMOS: version
CFI: Comparative Fit Index
TWM: Teacher Workload and Management
IPLD: Inclusion Benefits and Levels of Disability
LCIS: Learning Challenges in Inclusive Settings
ES: Excluded Students
PT: Professional Training
TLI: Tucker-Lewis Index
RMSEA: Root Mean Square Error of Approximation
AIC: Akaike Information Criterion
MCAR: Missing completely at random
DV: Discriminant validity
